# BiasAway: command-line and web server to generate nucleotide composition-matched DNA background sequences

**DOI:** 10.1093/bioinformatics/btaa928

**Published:** 2020-11-02

**Authors:** Aziz Khan, Rafael Riudavets Puig, Paul Boddie, Anthony Mathelier

**Affiliations:** Centre for Molecular Medicine Norway (NCMM), Nordic EMBL Partnership, University of Oslo, 0349 Oslo, Norway; Stanford University School of Medicine, Stanford Cancer Institute, Stanford, CA 94304, USA; Centre for Molecular Medicine Norway (NCMM), Nordic EMBL Partnership, University of Oslo, 0349 Oslo, Norway; Centre for Molecular Medicine Norway (NCMM), Nordic EMBL Partnership, University of Oslo, 0349 Oslo, Norway; Centre for Molecular Medicine Norway (NCMM), Nordic EMBL Partnership, University of Oslo, 0349 Oslo, Norway; Department of Medical Genetics, Oslo University Hospital, 0424 Oslo, Norway

## Abstract

**Motivation:**

Accurate motif enrichment analyses depend on the choice of background DNA sequences used, which should ideally match the sequence composition of the foreground sequences. It is important to avoid false positive enrichment due to sequence biases in the genome, such as GC-bias. Therefore, relying on an appropriate set of background sequences is crucial for enrichment analysis.

**Results:**

We developed BiasAway, a command line tool and its dedicated easy-to-use web server to generate synthetic sequences matching any k-mer nucleotide composition or select genomic DNA sequences matching the mononucleotide composition of the foreground sequences through four different models. For genomic sequences, we provide precomputed partitions of genomes from nine species with five different bin sizes to generate appropriate genomic background sequences.

**Availability and implementation:**

BiasAway source code is freely available from Bitbucket (https://bitbucket.org/CBGR/biasaway) and can be easily installed using bioconda or pip. The web server is available at https://biasaway.uio.no and a detailed documentation is available at https://biasaway.readthedocs.io.

**Supplementary information:**

Supplementary data are available at *Bioinformatics* online.

## 1 Introduction

Transcription factors (TFs) are proteins that control cellular processes by binding to DNA in a sequence specific manner to modulate gene expression (Lambert *et al.*, 2018). In gene regulation studies, motif enrichment analyses have been key to identify TF binding sites in regulatory regions. Accurate motif enrichment analysis depends on background DNA sequences that represent an adequate null hypothesis (Boeva, 2016; Simcha *et al.*, 2012; Worsley Hunt *et al.*, 2014). Indeed, genomes do not harbor a uniform sequence/nucleotide composition but contain local sequence biases such as variation of GC content (Badis *et al.*, 2009; Nekrutenko *et al.*, 2000; Plotkin *et al.*, 2011; Worsley Hunt *et al.*, 2014). Therefore, selection of background sequences has a strong influence on motif enrichment analysis. Ideally, background sequences need to match the foreground sequence compositional features to perform accurate enrichment analyses.

The importance of DNA background sequences for motif over-representation analysis has recurrently been highlighted (Boeva, 2016; Mariani *et al.*, 2017; Simcha *et al.*, 2012; Worsley Hunt *et al.*, 2014) and several approaches have been developed to address this problem. A classical approach consists in randomly shuffling foreground sequences to preserve mono- or di-nucleotide compositions to reduce nucleotide composition biases (Jiang *et al.*, 2008; Roadmap Epigenomics Consortium *et al.*, 2015; Weirauch *et al.*, 2014). Tools such as HOMER (Heinz *et al.*, 2010), RSAT (Nguyen *et al.*, 2018; Thomas-Chollier *et al.*, 2008) and GENRE (Mariani *et al.*, 2017) offer the possibility to generate sequences that are either synthetic or genomic. Nevertheless, none offers multiple approaches or models to construct synthetic and genomic background sequences matching the nucleotide composition of foreground sequences in a unified framework.

We previously developed BiasAway, a command-line tool with six distinct methodologies to generate DNA background sequences (Worsley Hunt *et al.*, 2014). Background sequences generated by BiasAway can either be synthetic or real genomic sequences that match the global or local mono- or di-nucleotide composition of user-provided sequences.

We updated BiasAway to generate synthetic sequences matching any k-mer nucleotide composition or select genomic DNA sequences matching the mononucleotide composition of the foreground sequences. BiasAway is now developed with Python-3 and can easily be installed through bioconda and pip. Finally, we implemented a web server companion, which comes with precomputed genomic partitions with five different bin sizes from nine species to generate background sequences. BiasAway is open source and its source code and interactive web interface are freely available at https://biasaway.uio.no.

## 2 Results

### 2.1 BiasAway background models

BiasAway provides flexibility to the user to choose from the four models (modules) to generate synthetic or real genomic background sequences that conserve either the global and/or local nucleotide composition of the foreground sequences. Specifically, the four approaches generate background sequences through (i) k-mer shuffling of the foreground sequences, (ii) k-mer shuffling of the foreground sequences using a sliding window, or extracting real genomic sequences matching (iii) the global mononucleotide composition or (iv) the local mononucleotide composition distribution (using a sliding window) of the foreground sequences. To match local nucleotide composition, BiasAway utilizes a sliding window over the input sequences to determine the %GC distribution along them and find background sequences with similar distribution. Altogether, BiasAway is a unique unified framework to generate synthetic or genomic DNA sequences [supporting the IUPAC alphabet (IUPAC-IUB Commission on Biochemical Nomenclature (CBN), 1970)] with more features than existing tools (Heinz *et al.*, 2010; Mariani *et al.*, 2017; Nguyen *et al.*, 2018), such as a variety of models, a web interface, a large number of pre-computed genomic sequences and an easy command-line installation (Supplementary Table S1).

#### 2.1.1 Synthetic k-mer shuffled sequences

This model permutes the nucleotides of the target sequences by keeping any k-mer composition of the original sequence selected by the user. For instance, the user can select *k* = 2 to preserve dinucleotide composition, which would conserve CpG distributions. BiasAway relies on the uShuffle python package to shuffle the provided sequences (Jiang *et al.*, 2008). This module should be run when the user aims at preserving the global k-mer nucleotide frequencies of input sequences. To read the help of this module, the user can type: *biasaway k.*

#### 2.1.2 Synthetic k-mer shuffled sequences in a sliding window

This approach is based on a sliding window to consider sub-regions of distinct nucleotide composition within the input sequences, which could be derived from evolutionary changes such as insertion of repetitive sequences, local rearrangements or biochemical missteps (see module 4 as well). The model generates a background sequence by shuffling the nucleotides within a sliding window *W* (default 100 bp) with a step S (default 50 bp) to conserve the local k-mer nucleotide composition for each sequence in the target sequences. This module should be run when the user aims at preserving the local k-mer nucleotide frequencies of input sequences. To read the help of this module, type: *biasaway w.*

#### 2.1.3 Genomic mononucleotide distribution matched sequences

This model requires both foreground and a set of genomic background sequences to be drawn as input. We also provide several background options for multiple species to choose from. First, GC composition of each target sequence is computed and sequences are assigned to bins in steps of 1% GC and the same is applied to the background pool of sequences. Then for each target sequence in a given GC bin, it randomly selects a background sequence from the equivalent background 1% GC bin. This module should be run when the user aims at selecting genuine genomic background sequences from a pool of provided genomic sequences to match the distribution of mononucleotide for each target sequence. To read the help of this module, type: *biasaway g.*

#### 2.1.4 Genomic mononucleotide distribution within a sliding window matched sequences

This method requires both foreground and a set of genomic background sequences to be drawn as input. It first computes the distribution of %GC composition within a sliding window *W* (default 100 bp) with a step *S* (default 50 bp) for each sequence in the input set of target sequences. Then it matches each target sequence to a background sequence with a similar %GC distribution (mean ± SD stdev over the sliding windows, default SD = 2.6) (Worsley Hunt *et al.*, 2014). This module should be run when the user aims at selecting genuine genomic background sequences from a pool of provided genomic sequences to match the local distribution of mononucleotide for each target sequence. To read the help of this module, type: *biasaway c.*

### 2.2 Quality control plots and metrics

**Fig. 1.**
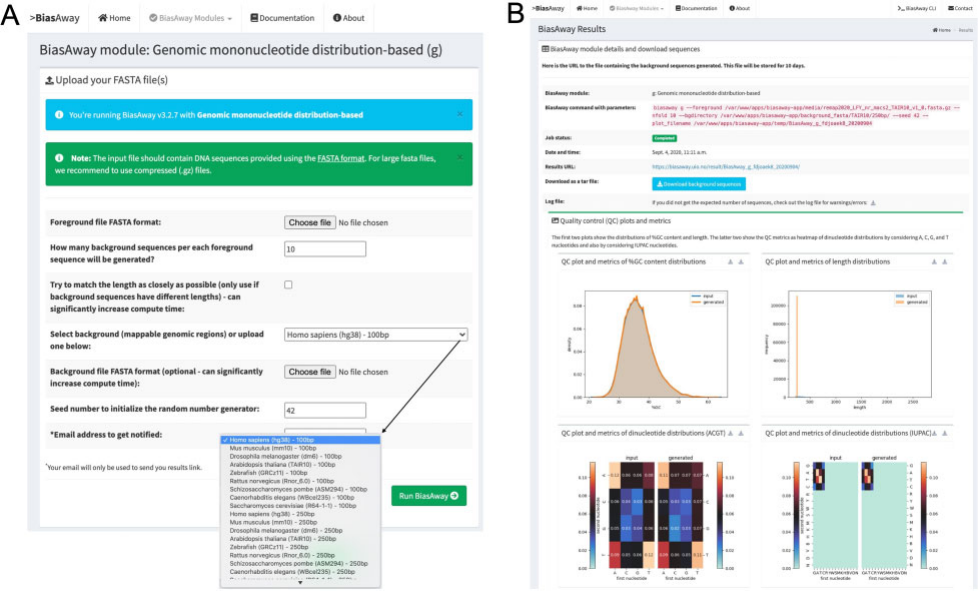
Screenshots of the BiasAway web application when launching the module [*g*] (**A**) and the corresponding result page (**B**)

BiasAway provides quality control (QC) plots and metrics to assess the similarity of the mono- and di-nucleotide, and length distributions for the foreground and background sequences (Fig. 1). Specifically, four plots are provided to visualize how similar the foreground and background sequences are when considering (i) their distributions of %GC content using density plots, (ii) their dinucleotide contents considering all IUPAC nucleotides using a heatmap, (iii) their dinucleotide contents considering adenine, cytosine, guanine and thymine nucleotides using a heatmap and (iv) their distributions of lengths. For each of the four QC plots, BiasAway provides QC metrics corresponding to the mean absolute error [implemented in scikit-learn (Pedregosa *et al.*, 2011)] and goodness of fit [implemented in scipy (Virtanen *et al.*, 2020)] computed as Pearson's chi-squared statistic, log-likelihood ratio test (G-test) (McDonald, 2014; Sokal *et al.*, 1981), and the Cressie-Read power divergence (Cressie *et al.*, 1984).

### 2.3 BiasAway command-line and web server

The BiasAway tool is implemented in Python 3, is open source (https://bitbucket.org/CBGR/biasaway), and can easily be installed using bioconda (Grüning *et al.*, 2018) or pip. A detailed documentation is provided at http://biasaway.readthedocs.io/ (available as Supplementary Text).

For online generation of background sequences and to help non-programmers, we provide an interactive and easy-to-use web interface for BiasAway. The web server is developed using the Django MVC framework Django and Bootstrap for user interface and is available at http://biasaway.uio.no (Fig. 1). The web server comes with precomputed genomic partitions of 100, 250, 500, 750 and 1000 bp bins for the genome of nine species (*Arabidopsis thaliana*; *Caenorhabditis elegans*; *Danio rerio*; *Drosophila melanogaster*; *Homo sapiens*; *Mus musculus*; *Rattus norvegicus*; *Saccharomyces cerevisiae*; and *Schizosaccharomyces pombe*; Fig. 1A). The background sequences are provided to the users through Zenodo (https://doi.org/10.5281/zenodo.3923866) and were generated using the script available at https://bitbucket.org/CBGR/biasaway_background_construction, which can be run by users to generate their own background sequences. The result page provides the QC plots computed from the provided and generated sequences for comparison (Fig. 1B).
